# Ki-67 can be used for further classification of triple negative breast cancer into two subtypes with different response and prognosis

**DOI:** 10.1186/bcr2834

**Published:** 2011-03-02

**Authors:** Bhumsuk Keam, Seock-Ah Im, Kyung-Hun Lee, Sae-Won Han, Do-Youn Oh, Jee Hyun Kim, Se-Hoon Lee, Wonshik Han, Dong-Wan Kim, Tae-You Kim, In Ae Park, Dong-Young Noh, Dae Seog Heo, Yung-Jue Bang

**Affiliations:** 1 Department of Internal Medicine, Seoul National University College of Medicine, 101 Daehang-ro, Jongno-gu, Seoul, 110-744, Korea; 2 Cancer Research Institute, Seoul National University College of Medicine, 101 Daehang-ro, Jongno-gu, Seoul, 110-744, Korea; 3 Department of Surgery, Seoul National University College of Medicine, 101 Daehang-ro, Jongno-gu, Seoul, 110-744, Korea; 4 Department of Pathology, Seoul National University College of Medicine, 101 Daehang-ro, Jongno-gu, Seoul, 110-744, Korea

## Abstract

**Introduction:**

Triple negative breast cancer (TNBC) has a poorer survival, despite a higher response rate to neoadjuvant chemotherapy. The purpose of this study was to identify the predictive or prognostic value of Ki-67 among patients with TNBC treated with neoadjuvant chemotherapy, and the role of Ki-67 in further classification of TNBC.

**Methods:**

A total of 105 TNBC patients who received neoadjuvant docetaxel/doxorubicin chemotherapy were included in the present study. Pathologic complete response (pCR) rate, relapse-free survival (RFS), and overall survival (OS) were compared according to the level of Ki-67.

**Results:**

pCR was observed in 13.3% of patients. TNBC with high Ki-67 expression (≥10%) showed a higher pCR rate to neoadjuvant chemotherapy than TNBC with low Ki-67 expression. None of the low Ki-67 group achieved pCR (18.2% in the high Ki-67 group *vs*. 0.0% in the low Ki-67 group, *P *= 0.019). However, a high Ki-67 expression was significantly associated with poor RFS and OS in TNBC, despite a higher pCR rate (*P *= 0.005, *P *= 0.019, respectively). In multivariate analysis, high Ki-67 was an independent prognostic factor for RFS in TNBC (hazard ratio = 7.82, *P *= 0.002). The high Ki-67 group showed a similar pattern of recurrence with overall TNBC, whereas the low Ki-67 group demonstrated a relatively constant hazard rate for relapse.

**Conclusions:**

TNBC with high Ki-67 was associated with a more aggressive clinical feature despite a higher pCR rate. High proliferation index Ki-67 can be used for further classification of TNBC into two subtypes with different responses and prognosis.

## Introduction

Triple negative breast cancer (TNBC) demonstrates poor prognosis because of aggressive tumor biology, and lack of targeted agents such as trastuzumab or tamoxifen [1,2]. TNBC has a pattern of rapid recurrence following diagnosis, and the peak risk of recurrence is within three years [3,4]. However, after the peak risk period, the risk of recurrence declines rapidly, and recurrences seldom occur thereafter [3,4].

Several reports suggested that TNBC was a heterogeneous group comprising subtypes with different clinical outcomes, and further classification of TNBC using cytokeratin (CK) 5/6 and epidermal growth factor receptor (EGFR) was useful to discriminate these subtypes [5-7]. True basal subtype in TNBC, which was defined as CK5/6 positive or EGFR positive, has shown poorer survival than CK5/6 and EGFR negative TNBC, which meant that TNBC could be divided into two subtypes: the aggressive clone and the less aggressive clone. New clinically applicable biologic markers for TNBC need to be developed in order to identify the patients with poor prognosis, and alternative treatment options are needed [1].

The proliferation marker Ki-67 has repeatedly been confirmed as an independent predictive and prognostic factor in early breast cancer [8]. Breast cancer with high Ki-67 expression responds better to chemotherapy [9-12], but is associated with poor prognosis [13-16]. This phenomenon is similar to the triple negative paradox, which denotes that TNBC had a poorer survival, despite a higher response rate to neoadjuvant chemotherapy [4,10,17]. In addition, TNBC is associated with a higher expression of Ki-67 than non-TNBC [10,18]. However, to date, the reason for the triple negative paradox is not clear, and there is little study focusing on the clinical significance of Ki-67 in TNBC. The purpose of this study was to identify the predictive or prognostic value of Ki-67 among patients with TNBC treated with neoadjuvant chemotherapy, and the role of Ki-67 in further classification of TNBC.

## Materials and methods

### Patients and chemotherapy

Recently, we conducted neoadjuvant docetaxel/doxorubicin chemotherapy in stage II or III breast cancer, and reported the prognostic and predictive role of the molecular markers [10,19]. The detailed eligibility criteria and regimen were described in our prior reports [10,19]. In brief, the patients received three cycles of neoadjuvant docetaxel/doxorubicin chemotherapy by intravenous infusion every three weeks. After three cycles of neoadjuvant chemotherapy, the patients were re-evaluated for response and underwent curative surgery. Subsequently, the patients received three more cycles of docetaxel/doxorubicin as an adjuvant chemotherapy, followed by hormonal or radiation therapy, if indicated [20]. Between January 2002 and September 2008, a total of 370 consecutive patients who received neoadjuvant docetaxel/doxorubicin chemotherapy at Seoul National University Hospital were included in the present study. Among the 370 patients, 109 patients were classified as TNBC. We excluded one patient with metaplastic carcinoma because triple negative phenotype in metaplastic carcinoma showed a different tumor biology than that of invasive ductal carcinoma [21]. Three patients were excluded because Ki-67 was not available due to lack of tissue. Finally, a total of 105 TNBC patients were analyzed. This study protocol was reviewed and approved by the Institutional Review Board of the Seoul National University Hospital (approval number: H-1003-058-313). Because this study was performed using a total of 370 consecutive patients in our database, and involved no more than minimal risk for the subjects, the Institutional Review Board approved our request for the waiver of informed consent. Recommendations of the Declaration of Helsinki for biomedical research involving human subjects were also followed.

### Immunohistochemistry

We performed an immunohistochemistry (IHC) using tissues obtained before treatment. Estrogen receptor (ER), progesterone receptor (PR), human epidermal growth factor receptor 2 (HER2), p53, bcl-2, and Ki-67 expressions were evaluated. IHC was performed as previously described [14,22]. ER and PR positivity was defined as ≥10% positive tumor cells with nuclear staining. HER2 positivity was defined as either HER2 gene amplification by fluorescent *in situ *hybridization or scored as 3+ by IHC [23]. In case of HER2 2(+), fluorescent *in situ *hybridization was performed to determine HER2 positivity. TNBC was defined as ER(-), PR(-), and HER2(-), regardless of the expression of EGFR and basal cytokeratins. Only cytoplasmic staining was scored as positive for bcl-2, regardless of the intensity of the stained cells. Cells stained for Ki-67 and p53 were counted and expressed as a percentage. The percentage was determined by the number of Ki-67 positive cells among the total number of counted tumor cells. High expression of Ki-67 was defined as ≥10%, because 10% as cutoff provided the best prognosis-prediction results in our institute [14]. Specimens with no residual invasive carcinoma in the both breast and lymph nodes were classified as pathologic complete response (pCR). Residual ductal carcinoma *in situ *was also included in the pCR category [24]. Otherwise the specimens which did not achieve pCR category were classified as residual disease

### Statistics

Relapse-free survival (RFS) was determined as the interval between the initiation of neoadjuvant chemotherapy and the date when disease relapse or progression was first documented, or the date of death from any cause. Overall survival (OS) was measured from the date neoadjuvant chemotherapy was initiated to the date of death.

The significance of the difference in the variables among two Ki-67 groups was calculated using Chi-square test or Fisher's exact test, where appropriate. The Kaplan-Meier product limit method and the Cox proportional hazard regression (PHR) model were used for survival analysis. The multivariate Cox PHR model was used to develop a prediction model for risk of relapse and death. Discrimination for survival data was evaluated using the C statistic with concordance index (C-index) [25,26], which is similar in concept to the area under the receiver operating characteristic (ROC) curve in the logistic model, but is appropriate for censored data. The C-index is the probability that given two subjects, one who will develop an event and the other who will not, the model will assign a higher probability of an event to the former [25]. In general, the model is considered as good for C-index value above 0.75.

The log-rank tests were used to compare RFS or OS between different groups. Hazard function is the instantaneous failure rate at time *t*, which is the probability of event in the next small interval. All statistical tests were two-sided, with the level of significance established at *P *< 0.05. Statistical analyses were performed using STATA statistical software version 11.0 (STATA, College Station, TX, USA) and R software version 2.10.1 [27]. R package with theDesign, survivalROC, and survcomp libraries.

## Results

Table 1 shows the baseline characteristics of the 105 patients. pCR was observed in 13.3% of the patients. With a median follow-up duration of 33.6 months, 33 relapse events occurred, and 20 patients died of disease progression. Estimated one-, two-, and three-year RFS rates, as calculated by the Kaplan-Meier method, were 83.8%, 71.6%, and 64.6%, respectively. The median value of Ki-67 was 20.0% (range = 0.0 to 80.0%, standard deviation = 23.3).

**Table 1:** Baseline characteristics of 105 patients

Characteristics	Total No. of Pt (%)	Low Ki-67 No. of Pt (%)	High Ki-67 ^a^ No. of Pt (%)	*P*-value
Median age (range)				
Age <35 years	18 (17.1)	5 (17.9)	13 (16.9)	0.907
Age ≥35 years	87 (82.9)	23 (82.1)	64 (83.1)	
Performance status				
ECOG 0	25 (23.8)	7 (25.0)	18 (23.4)	0.686
ECOG 1	78 (74.3)	21 (75.0)	57 (74.0)	
ECOG 2	2 (1.9)	0 (0.0)	2 (2.6)	
Pathologic characteristics				
Invasive ductal carcinoma	101 (96.2)	28 (100.0)	73 (94.8)	0.572
Others	4 (3.8)	0 (0.0)	4 (5.2)	
Initial clinical stage				
IIA	4 (3.8)	1 (3.6)	3 (3.9)	0.985
IIB	19 (18.1)	5 (17.9)	14 (18.2)	
IIIA	45(42.9)	11 (39.3)	34 (44.2)	
IIIB	18 (17.1)	5 (17.9)	13 (16.9)	
IIIC	19 (18.1)	6 (21.4)	13 (16.9)	
Inflammatory breast cancer				
No	97 (92.4)	25 (89.3)	72 (93.5)	0.437
Yes	8 (7.6)	3 (10.7)	5 (6.5)	
Type of surgery				
Breast conserving	44 (41.9)	13 (46.4)	31 (40.3)	0.571
Mastectomy	61 (58.1)	15 (53.6)	46 (59.7)	
Radiation therapy				
No	11 (10.5)	2 (7.1)	9 (11.7)	0.723
Yes	94 (89.5)	26 (92.9)	68 (88.3)	
Nuclear grade				
I	1(1.0)	0 (0.0)	1 (1.3)	0.260
II	7 (6.7)	2 (7.1)	5 (6.5)	
III	88 (73.3)	24 (85.7)	53 (68.8)	
Unknown	20 (19.0)	2 (7.1)	18 (23.4)	
Histologic grade				
I	0 (0.0)	0 (0.0)	0 (0.0)	0.097
II	23 (21.9)	10 (35.7)	13 (16.9)	
III	74 (70.5)	17 (60.7)	57 (74.0)	
Unknown	8 (7.6)	1 (3.6)	7 (9.1)	
bcl-2				
Negative	66 (62.9)	15 (53.6)	51 (66.2)	0.235
Positive	39 (37.1)	13 (46.4)	26 (33.8)	
p53				
Negative	39 (37.1)	12 (42.9)	27 (35.1)	0.657
Positive	65 (61.9)	16 (57.1)	49 (63.6)	
Unknown	1 (1.0)	0 (0.0)	1 (1.3)	
pCR				
No	91 (86.7)	28 (100.0)	86 (81.8)	0.019
Yes	14 (13.3)	0 (0.0)	14 (18.2)	

Abbreviations: ECOG, Eastern Cooperative Oncology Group; pCR, pathologic complete response.

^a ^High Ki-67 expression was defined as ≥10%

### Response to neoadjuvant chemotherapy by Ki-67 expression status in TNBC

Clinicopathologic characteristics between high and low Ki-67 groups were not different (Table 1). However, TNBC with high Ki-67 showed a higher pCR rate to neoadjuvant chemotherapy than TNBC with low Ki-67, and none of the patients in the low Ki-67 group achieved pCR (18.2% in the high Ki-67 group *vs*. 0.0% in the low Ki-67 group, *P *= 0.019). pCR rate was proportionally associated with the level of Ki-67. When analyzing with a cutoff of Ki-67 quartile (Q), the pCR rates were 0.0% in the first Q, 14.3% in the second Q, 18.2% in the third Q, and 22.2% in the fourth Q, respectively.

### Survival by Ki-67 expression status in TNBC

**Figure 1:**
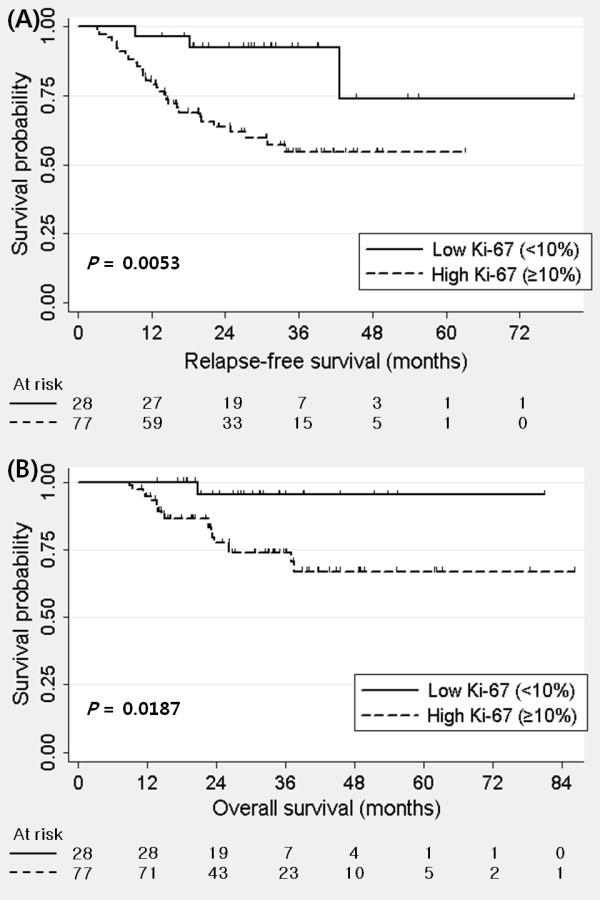
**Kaplan-Meier curve of (A) relapse-free survival and (B) overall survival by Ki-67 in triple negative breast cancer**.

**Figure 2:**
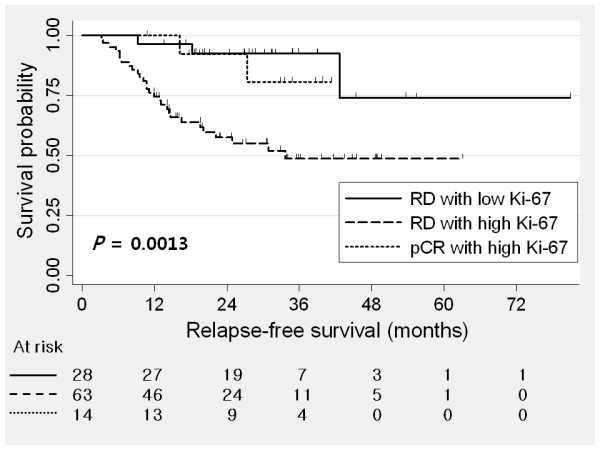
**Relapse-free survival (RFS) as a function of pathologic response**. Residual disease (RD) with high Ki-67 showed poorer RFS than RD with low Ki-67 and pathologic complete response (pCR) with high Ki-67.

High Ki-67 expression was significantly associated with poor RFS and OS in TNBC (Figure 1). Combining pCR and Ki-67, residual disease (RD) with high Ki-67 showed poorer RFS than RD with low Ki-67 and pCR with high Ki-67 (Figure 2). RD with low Ki-67 showed better RFS than RD with high Ki-67 (*P *= 0.017). However, there was no statistical difference in RFS between pCR with high Ki67 and RD with low Ki-67 (*P *= 0.449). Univariate analysis revealed that initial clinical stage, pathologic nodal stage, histologic grade, and Ki-67 were prognostic factors in TNBC. However, bcl2 and p53 were not associated with RFS. In multivariate analysis, Ki-67 was the independent prognostic factor for RFS in TNBC (Table 2). High Ki-67 expression was also significantly associated with poorer OS in multivariate analysis as well as univariate analysis (Table S1 in Additional file 1).

**Table 2:** Univariate and multivariate Cox regression analysis of factors associated with relapse-free survival in triple negative breast cancer

	Univariate			Multivariate		
Variables	HR	95% CI	*P*-value	HR	95% CI	*P*-value
Age						
<35 years	1			-		
≥35 years	0.77	0.33 to 1.76	0.529			
Performance status						
ECOG0	1			-		
ECOG1	1.41	0.54 to 3.70	0.483			
ECOG2	4.29	0.83 to 22.17	0.082			
Initial clinical stage						
IIA, IIB	1			1		
IIIA	9.75	1.30 to 73.30	0.027	7.51	0.98 to 57.83	0.053
IIIB	9.59	1.18 to 77.94	0.034	7.72	0.92 to 64.93	0.060
IIIC	18.59	2.37 to 146.04	0.005	14.75	1.74 to 125.11	0.014
Pathologic N stage						
pN0	1			1		
pN1	3.79	1.47 to 9.77	0.006	5.87	2.08 to 16.59	0.001
pN2	4.14	1.33 to 12.88	0.014	4.95	1.45 to 16.91	0.011
pN3	6.51	2.09 to 20.26	0.001	7.54	2.14 to 26.56	0.002
bcl-2						
Negative	1			-		
Positive	0.87	0.43 to 1.76	0.691			
p53						
Negative	1			-		
Positive	1.05	0.52 to 2.16	0.885			
Histologic grade						
II	1			1		
III	3.37	1.03 to 11.06	0.045	2.04	0.57 to 7.37	0.275
Ki-67						
Low Ki-67	1			1		
High Ki-67	4.64	1.41 to 15.22	0.011	7.82	2.18 to 28.13	0.002
Continuous ^a^	1.02	1.01 to 1.03	0.003	-		

Abbreviations: CI, confidence interval; HR, hazard ratio.

^a ^Entered as continuous variable.

**Figure 3:**
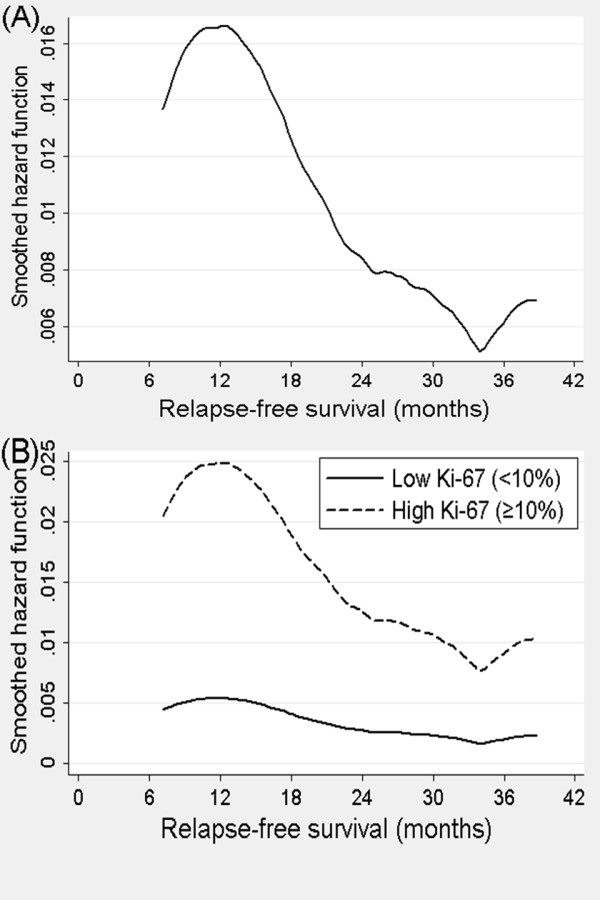
**Smoothed relapse hazard as shown by hazard function in TNBC**. **(A) **Hazard functions for relapse in all TNBC patients. **(B) **Hazard functions for relapse by Ki-67.

The discriminatory ability of the model for RFS was measured using C statistics. The C-index was 0.83 (95% confidence interval 0.78 to 0.89), indicating good model performance. We examined the hazard function for relapse. Figure 3A shows kernel estimates of the hazard functions of relapse in TNBC. TNBC has a pattern of rapid recurrence following diagnosis, and peak risk of recurrence was at 12 months. After three years, relapse did not occur except for one patient. When evaluating the hazard function by Ki-67 expression status, the high Ki-67 group showed a similar pattern of recurrence with overall TNBC, whereas the low Ki-67 group demonstrated a relatively constant hazard rate for relapse (Figure 3B).

## Discussion

In the present study, we found that TNBC with high Ki-67 expression had poorer survival than TNBC with low Ki-67 expression, despite a higher pCR rate. Furthermore, TNBC with high Ki-67 expression showed rapid recurrence within three years, whereas TNBC with low Ki-67 expression showed a near-constant recurrence rate. Ki-67 could divide TNBC into two different clinical subtypes.

It is well confirmed that pCR to neoadjuvant chemotherapy is an independent prognostic factor for survival [28-30]. However, several studies have shown that TNBC has a higher pCR rate but poorer survival than non-TNBC [4,10,17]. Like this triple negative paradox, we found that high Ki-67 in TNBC was associated with a higher pCR rate and poorer survival. High Ki-67, representing high proliferation potential, could explain the paradoxical feature.

Ki-67 is a cell proliferation-associated antigen that is expressed in all stages of the cell proliferative cycle except the G0 (quiescent) phase [31]. Among the proliferation-related markers, Ki-67 is known to be the simplest and a widely used method to assess tumor proliferation. Several studies have investigated the predictive and prognostic values of Ki-67 in breast cancer patients receiving neoadjuvant chemotherapy [9,11,32-39]. High Ki-67 was associated with higher response rate to neoadjuvant chemotherapy in breast cancer [9,11,34,35,37], although no association between high Ki-67 and response rate was also reported [33,36,39]. Some studies emphasized the change of Ki-67 or postoperative level of Ki-67 in predicting the response [9,32,33,37,38]. Recently, Jones *et al. *[9] reported that higher pre-treatment Ki-67 was significantly more likely to achieve pCR than lower Ki-67, but was associated with poor RFS and OS. This was consistent with our results. However, the role of Ki-67 is still not yet conclusive because of heterogeneous patient populations, small sample sizes, and different chemotherapeutic regimens in previous studies [9,11,33-39].

Generally, tumor responsiveness to chemotherapy is believed to be associated with longer survival. However, TNBC, which has a higher Ki-67 level than non-TNBC [10,18] showed a higher pCR rate with poor survival [4,10,17]. Tumor responsiveness might not always affect prolonged survival in a tumor with high Ki-67. We first found this paradoxical feature in TNBC, and our results suggest that further classification using Ki-67 levels might improve the prognostic significance of pCR in neoadjuvant chemotherapy. Surely, it is not yet certain whether Ki-67 itself is a causable indicator for the triple negative paradox or just a mediator for another unknown factor. Further research to find out direct association is warranted.

Previous reports indicated that TNBC had a pattern of early recurrence within the first three years of follow-up; however, the risk of recurrence significantly decreased thereafter [3,4]. Our study confirms these findings. Furthermore, when analyzing hazard rate by Ki-67 status, only TNBC with high Ki-67 demonstrated a pattern of early recurrence, whereas the low Ki-67 subgroup did not show any pattern at all. This suggests that an early recurrence pattern of TNBC is ascribed to the high Ki-67 subgroup which has a high proliferation potential. TNBC seems to be a heterogeneous group with at least two different clinical courses. TNBC with high proliferation potential should be followed-up more frequently within three years, and could be a candidate for additional postoperative treatments with different mechanisms. Additional discriminating markers should be sought to further refine the classification of TNBC.

The present study has some limitations. First, we did not examine basal markers, namely, CK5/6 and EGFR, which are potential classifiers that differentiate TNBC into an aggressive basal clone and a less aggressive non-basal clone [40,41]. Further research is needed to determine which will be a better classifier for TNBC among Ki-67 and basal markers. Second, the pCR rate of our study (13.3%) was relatively lower than that of another study [17]. This was because only three cycles of neoadjuvant chemotherapy were performed and the tumor size was relatively large.

## Conclusions

In conclusion, TNBC with high Ki-67 was associated with a more aggressive clinical feature despite a higher pCR rate. Ki-67 could explain the triple negative paradox, and Ki-67 can be used for further classification of TNBC into two subtypes with different prognosis. Our report suggests that TNBC with residual disease and high Ki-67 expression should be a candidate for additional postoperative treatment such as platinum-based chemotherapy, or clinical trials specifically testing novel therapies in order to improve the outcome for this high-risk group of patients. In addition, TNBC with high Ki-67 should be followed-up more frequently within three years to guard for any recurrence.

## Abbreviations

C-index: C statistic with concordance index; CK: cytokeratin; EGFR: epidermal growth factor receptor; ER: estrogen receptor; HER2: human epidermal growth factor receptor 2; IHC: immunohistochemistry; OS: overall survival; pCR: pathologic complete response; PHR: proportional hazard regression; PR: progesterone receptor; Q: quartile; RD: residual disease; RFS: relapse-free survival; ROC: receiver operating characteristic; TNBC: triple negative breast cancer.

## Competing interests

The authors declare that they have no competing interests.

## Authors' contributions

SAI and BK designed the concept of the study. SAI, SWH, DYO, JHK, SHL, DWK, TYK, DSH and YJB were responsible for provision of study patients and chemotherapy. DYN and WH were responsible for provision of study patients and surgery. IAP was responsible for pathologic examination and immunohistochemistry. BK and KHL were responsible for data gathering, statistical analysis and interpretation. SAI and BK wrote the final manuscript. All authors read and approved the final manuscript.

## Supplementary Material

Additional file 1 **Supplementary Table S1**. Univariate and multivariate Cox regression analysis of factors associated with overall survival in triple negative breast cancer. Click here for file
