# Supplementary material for: Ki-67 can be used for further classification of triple negative breast cancer into two subtypes with different response and prognosis
Source: Breast Cancer Res. 2011 Mar 2;13(2):R22. doi: 10.1186/bcr2834 (PMC3219180; doi:10.1186/bcr2834)
Supplement: Additional file 1 — Supplementary Table S1. Univariate and multivariate Cox regression analysis of factors associated with overall survival in triple negative breast cancer. [file bcr2834-S1.DOC]

Table S1. **Univariate and multivariate Cox regression analysis of factors associated with overall survival in triple negative breast cancer**

|  | Univariate | | | Multivariate | | |
| --- | --- | --- | --- | --- | --- | --- |
| Variables | HR | 95% CI | *P*-value | HR | 95% CI | *P*-value |
| Age  <35  ≥35 | 1  0.82 | 0.27-2.45 | 0.718 | - |  |  |
| Performance status  ECOG0  ECOG1  ECOG2 | 1  0.76  2.64 | 0.25-2.34  0.29-23.65 | 0.634  0.386 | - |  |  |
| Initial clinical stage  IIA, IIB, IIIA  IIIB  IIIC | 1  1.29  4.89 | 0.34-4.86  1.87-12.78 | 0.710  0.001 | 1  1.31  2.74 | 0.331-5.15  0.97-7.71 | 0.704  0.057 |
| Pathologic N stage  pN0  pN1  pN2  pN3 | 1  5.25  9.92  18.73 | 1.09-25.33  1.92-51.23  3.74-93.66 | 0.034  0.006  <0.001 | 1  7.41  10.59  24.41 | 1.52-36.09  1.97-56.95  4.35-136.96 | 0.013  0.006  <0.001 |
| bcl-2  Negative  Positive | 1  0.64 | 0.25-1.66 | 0.357 | - |  |  |
| p53  Negative  Positive | 1  1.42 | 0.55-3.70 | 0.470 | - |  |  |
| Histologic grade  II  III | 1  1.673 | 0.49-5.71 | 0.412 | - |  |  |
| Ki-67  Low Ki-67  High Ki-67  Continuous ^a^ | 1  7.69  1.02 | 1.03-57.47  1.00-1.04 | 0.047  0.034 | 1  15.73  - | 2.06-119.94 | 0.008 |

Abbreviations: HR, hazard ratio; CI, confidence interval.

^a^ Entered as continuous variable.
